# CANCER SURVIVORSHIP, AGING, AND SEXUAL HEALTH: GENDERED EXPERIENCES OF SEXUAL BEHAVIOR AND BODY IMAGE

**DOI:** 10.1093/geroni/igac059.2246

**Published:** 2022-12-20

**Authors:** Spencier Ciaralli, Gary Deimling, Dyanna Burnham, Colleen Kavanagh

**Affiliations:** Augustana University, Sioux Falls, South Dakota, United States; Case Western Reserve University, Cleveland, Ohio, United States; Case Western Reserve University, Cleveland, Ohio, United States; Case Western Reserve University, Cleveland, Ohio, United States

## Abstract

Previous research on aging has studied sexual health as well as cancer. However, less is known about the intersections of cancer survivorship, aging, and sexual health, particularly as it pertains to the gendered experiences of body image. The select studies available that consider the role of sex and intimacy after cancer have provided wholly quantitative or qualitative analyses. This study provides a mixed-methods approach of cancer survivorship and aging as it pertains to sexual health, particularly examining prostate, colorectal, and breast cancer survivors. In their narratives as to how cancer has influenced their sexual behavior, female breast cancer survivors construct their experiences around body image satisfaction. Men who are prostate cancer survivors note struggles around “performance” and impotence. Common issues for both men and women with colorectal cancer survivorship identify body image and impotence as affecting their sexual behavior. An underlying theme throughout all aging cancer survivors’ narratives is the role of relationships and the perceptions of their partner. Race, marital status, and education are also examined. The data analyzed are from an NCI funded, 10-year longitudinal study based on in-person interviews with 321 cancer survivors. The findings illustrate the importance of medical professionals providing wholistic after-care that is inclusive of aging survivors’ sexual health, relationships, and perception of self.

